# ChatGPT and generative AI in urology and surgery—A narrative review

**DOI:** 10.1002/bco2.390

**Published:** 2024-07-25

**Authors:** Shane Qin, Bodie Chislett, Joseph Ischia, Weranja Ranasinghe, Daswin de Silva, Jasamine Coles‐Black, Dixon Woon, Damien Bolton

**Affiliations:** ^1^ Department of Urology Austin Health Heidelberg Victoria Australia; ^2^ Department of Surgery University of Melbourne, Austin Health Melbourne Victoria Australia; ^3^ Department of Anatomy and Developmental Biology Monash University Melbourne Victoria Australia; ^4^ Department of Urology Monash Health Melbourne Victoria Australia; ^5^ Research Centre for Data Analytics and Cognition La Trobe University Melbourne Victoria Australia

**Keywords:** ChatGPT, generative artificial intelligence, machine learning, surgery, urology

## Abstract

**Introduction:**

ChatGPT (generative pre‐trained transformer [GPT]), developed by OpenAI, is a type of generative artificial intelligence (AI) that has been widely utilised since its public release. It orchestrates an advanced conversational intelligence, producing sophisticated responses to questions. ChatGPT has been successfully demonstrated across several applications in healthcare, including patient management, academic research and clinical trials. We aim to evaluate the different ways ChatGPT has been utilised in urology and more broadly in surgery.

**Methods:**

We conducted a literature search of the PubMed and Embase electronic databases for the purpose of writing a narrative review and identified relevant articles on ChatGPT in surgery from the years 2000 to 2023. A PRISMA flow chart was created to highlight the article selection process. The search terms ‘ChatGPT’ and ‘surgery’ were intentionally kept broad given the nascency of the field. Studies unrelated to these terms were excluded. Duplicates were removed.

**Results:**

Multiple papers have been published about novel uses of ChatGPT in surgery, ranging from assisting in administrative tasks including answering frequently asked questions, surgical consent, writing operation reports, discharge summaries, grants, journal article drafts, reviewing journal articles and medical education. AI and machine learning has also been extensively researched in surgery with respect to patient diagnosis and predicting outcomes. There are also several limitations with the software including artificial hallucination, bias, out‐of‐date information and patient confidentiality.

**Conclusion:**

The potential of ChatGPT and related generative AI models are vast, heralding the beginning of a new era where AI may eventually become integrated seamlessly into surgical practice. Concerns with this new technology must not be disregarded in the urge to hasten progression, and potential risks impacting patients' interests must be considered. Appropriate regulation and governance of this technology will be key to optimising the benefits and addressing the intricate challenges of healthcare delivery and equity.

## 1 INTRODUCTION

ChatGPT (generative pre‐trained transformer [GPT]) is the flagship model of generative artificial intelligence (AI), the new generation of AI that formulates responses to questions and prompts from human operators based on the familiarity of learned co‐occurrences of billions of data points. In the case of ChatGPT, these data points are text documents that contain much of written human knowledge while generative AI models trained on images, videos, audio recordings and programming code are also highly effective. As the fastest‐growing consumer application in human history, reaching a hundred million active users 2 months after its launch, ChatGPT is primed for application across every aspect of healthcare, including public health, diagnosis, treatment, disease management, academic research and clinical trials.^1^

In the machine learning space, the growth of large language models (LLMs) has accelerated since the release of the transformer model in 2017. Google introduced the Bidirectional Encoder Representations from Transformers (BERT), and OpenAI developed GPT‐1, 2, 3 and 4 with increasingly larger datasets and models as a result. For instance, GPT‐2 had 1.5 billion parameters and GPT‐3 was trained on 175 billion parameters. This was then fine‐tuned into GPT‐3.5 by introducing barriers to toxic content and packaged into a consumer‐friendly version resulting in ChatGPT.^2^ GPT‐4, which was released in March 2023, reportedly has 1.7 trillion parameters and has availability on a paid subscription service ChatGPT Plus. The conversational intelligence of ChatGPT stems from the formative capabilities of the additional layer of reinforcement learning from human feedback.^3^ These capabilities are chat, open Q&A, closed Q&A, text classification, text summarisation, text generation, information extraction, text rewrite, and brainstorming. LLMs are extremely expensive to develop and deploy, with this capability feasible only for large corporations. Multinational technology companies such as Google and Microsoft have hurriedly released their own chatbots in response to the success of ChatGPT. Microsoft has released Copilot (previously known as Bing Chat) and Google's own version is named Bard, which runs on Pathways Language Model (PaLM)‐2, its latest developed LLM.

In the urology sphere, there has been much research into AI in recent years. Various machine learning models including artificial neural networks (ANN), convolutional neural networks (CNN), natural language processing and computer vision have been utilised in various fields within urology. An ANN is a network of individual units that act as artificial neurons similar to that of a human brain. ANN has been used in uro‐oncology such as the prediction of prostate biopsy results and prediction of renal cancer recurrence after surgery. CNN is used for image and video recognition, analysis and classification. This network has been applied to prostate cancer pathology slides and cystoscopy images to improve the diagnosis of bladder cancer. Generative AI however is an area of AI that has taken the spotlight since the release of ChatGPT with significant investment being made to integrate it into healthcare. This article provides a comprehensive narrative review of AI and more recently generative AI, in urology and more broadly in surgery, and the various ways it is being used.

## 2 METHODS

**FIGURE 1:**
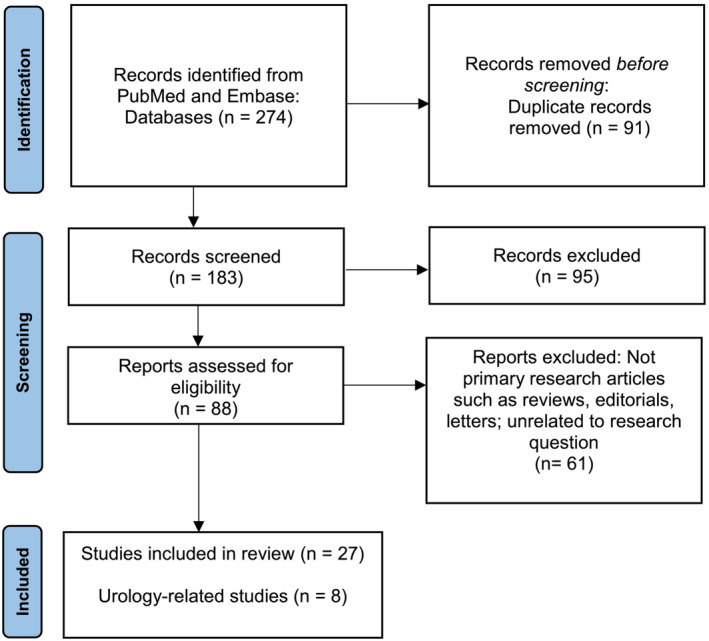
PRISMA flow chart.

We conducted a literature search of the PubMed and Embase electronic databases for the purpose of writing a narrative review and identified relevant articles on ChatGPT in surgery from the years 2000 to 2023. A PRISMA flow chart was created to highlight the article selection process. The search terms ‘ChatGPT’ and ‘surgery’ were intentionally kept broad given the nascency of the field. Studies unrelated to these terms were excluded. The results of the literature search were downloaded into Zotero software. Duplicate articles were removed (Figure 1).

## 3 DISCUSSION

### 3.1 Current use of AI in surgery

There has been research into AI and machine learning in surgery in recent years, however nothing similar to ChatGPT has been used before. Machine learning has been used to aid diagnosis in many surgical conditions including head and neck malignancies, vestibular and sensorineural hearing disorders, facial deformities, vascular conditions including aortic aneurysm/dissection and carotid stenosis, gynaecological malignancies, and also ophthalmic pathology including keratoconus, glaucoma and diabetic retinopathy.^4^, ^5^, ^6^, ^7^, ^8^ AI has also been used to predict patient outcomes in multiple areas including brain tumour and spinal surgery, readmissions and mortalities post‐cardiothoracic surgery, and probability of seizure freedom after paediatric epilepsy surgery.^9^, ^10^, ^11^, ^12^, ^13^

AI also has been used in pre‐operative planning in neurosurgery for minimally invasive approaches for tumour resection.^14^ AI‐assisted imaging has also found applications in detecting brain tumour volume and subtypes more accurately, fracture and breast cancer diagnosis, and image segmentation in aortic aneurysm/dissection and carotid stenosis.^6^, ^11^, ^14^ AI has also assisted in surgeries including robot support in neurosurgery, supervised robot suturing and bowel anastomosis in general surgery.^9^, ^11^ AI has additionally been used to optimise hearing aid and speech enhancement technologies in otolaryngology, and implant identification and gait analysis in orthopaedic surgery (see Table 1).^15^, ^16^

### 3.2 Use of AI in urology

Machine learning has been applied to many areas within urology, especially in genitourinary malignancies where diagnosis has been an area of focus. For diagnosing prostate cancers, two main areas include radiomics of multiparametric magnetic resonance imaging (MRI) and AI‐assisted analysis of histopathology.^17^ Other reported functions include diagnosis of urothelial cancer via radiomics and analysis of serum and urinary biomarkers. Studies in the diagnosis of renal cancers have similarly looked at computed tomography (CT) scans and serum metabolite biomarkers.^18^ AI has been used for predicting recurrence in prostate and bladder cancers, assessment of urolithiasis, and surgical skills evaluation (see Table 1).^18^, ^19^

Noteworthy given the exponential increase in AI utilisation and research is the lack of standardised reporting and explanation of systems when applying machine learning. As such, the Standardised Reporting of Machine Learning Applications in Urology (STREAM‐URO) framework has been developed.^20^ This has the potential to rapidly escalate the utility of AI in urologic practice beyond that of other areas of surgical practice.

**TABLE 1:** AI uses in surgery.

AI uses	Specialty	Examples
Diagnosis	Otolaryngology head and neck surgery	Head and neck cancers, vestibular disorders, sensorineural hearing loss prediction^4^
	Plastic surgery	Burns, congenital/acquired facial deformities, cosmetic surgery^5^
	Urology	AI‐assisted analysis of pathology slides, analysis of biomarkers and urine studies in prostate cancer, urinary metabolic markers in bladder cancer and serum metabolite biomarkers in renal cancer^17^, ^18^, ^19^
	Vascular surgery	Aortic aneurysm/dissection, carotid stenosis, peripheral artery disease, diabetic foot ulcer, venous disease, renal artery stenosis^6^
	Gynaecology	Gynaecological malignancies^7^
	Ophthalmology	Keratoconus, glaucoma, diabetic retinopathy^8^
Prognosis/predicting patient outcomes	Neurosurgery	Survival, recurrence, adverse events post‐surgery in brain tumour and spinal surgery^9^
	Cardiothoracic surgery	Readmissions and mortalities^10^
	General surgery	Multiple AI tools developed that predict post‐operative complications including post‐operative shock^11^
	Paediatric surgery	Predicting risk of clinical deterioration post paediatric cardiac surgery, and probability of seizure freedom after paediatric epilepsy surgery^12^, ^13^
	Orthopaedic surgery	
	Urology	Predicting recurrence in prostate and bladder cancers^18^, ^19^
Pre‐operative planning	Neurosurgery	Planning minimally invasive approaches for tumour resection^14^
Imaging	Neurosurgery	Radiomics to detect brain tumour volumes and subtypes more accurately^14^
	General surgery	Breast cancer diagnosis^11^
	Orthopaedic surgery	Bone fracture diagnosis^16^
	Urology	Radiomics of multiparametric prostate MRIs in prostate cancer, bladder cancer, renal cancer^17^, ^18^, ^19^
Robotic surgery	Neurosurgery	Robotic support^9^
	General surgery	Supervised robots suturing, bowel anastomosis^11^
Equipment/technology	Otolaryngology head and neck surgery	Optimisation of hearing aid technology, speech enhancement technologies^15^
	Orthopaedic surgery	Implant identification, gait analysis^16^

### 3.3 Use of chatbots in urology

There are many potential applications of ChatGPT for urologists that extend from its formative capabilities of text classification, summarisation and generation, plus information extraction. Other uses for ChatGPT include assisting in administrative tasks integrated into contemporary clinical workflow. Virtual assistants have been used to answer frequently asked questions regarding post‐operative care, provide personalised resources to patients and explain medical concepts in a simplified manner, plus write operation reports and discharge summaries based on data provided to it. Additionally, ChatGPT may be applied to medical student and resident education by generating educational materials on a broad range of topics (see Table 2).^21^ A study by Eppler et al. performed a survey of urologists on the experience with AI and utilisation of LLMs in research and clinical practice worldwide. They found that 47.7% of respondents used ChatGPT or another LLM in academia, but only 19.8% utilised a form of generative AI in clinical practice. The majority (62.2%) of urologists believe that potential ethical issues can arise from using ChatGPT in academic writing.^22^ Our group has used AI technology in developing similar chatbots in urologic surgery. The Patient Reported Information Multidimensional Exploration (PRIME) framework is a machine learning platform that has been used to extract and analyse information from Online Cancer Support Groups (OCSG). The PRIME AI framework has been used on prostate cancer patients to investigate emotions, treatment side effects and quality of life outcomes, which showed that patient emotions were overlooked especially in the younger cohort and their partners.^23^ A subsequent study assessed psychological morbidity in prostate cancer patients using PRIME. The results showed that participation in OCSG by patients led to a decrease in psychological stress and long‐term involvement led to an increase in emotional wellbeing.^24^ PRIME‐2 (PRIME version 2) also analysed patient decision‐making, functional and emotional outcomes in men undergoing robotic‐assisted laparoscopic prostatectomy (RARP) or open radical prostatectomy (ORP) from OCSG discussions.

The PRIME‐2 found similar side effect profiles between the two groups but detected greater negative emotions in the ORP group particularly in the post‐operative period due to pain in the first and third months and at 9 months from fear and anxiety of approaching PSA tests.^25^ PRIME was further applied on cancer discussion, including urologic cancers, on social media platforms during the COVID‐19 pandemic. It was shown to be able to detect emotions in real time with an increase in negative emotions coinciding with the beginning of the pandemic as well as a sharp rise in social media usage.^26^

Coskun et al. tested the usefulness of ChatGPT in providing patient information on prostate cancer by using quantifiable metrics to measure the accuracy, similarity and quality of information. It achieved this by using F1, precision and recall scores to measure accuracy. Cosine similarity was used to evaluate similarity, and a 5‐point Likert scale called the general quality score (GQS) assessed the quality. Two urologists independently graded the accuracy ChatGPT by comparing the answers generated by the model with a reference material. The average F1 score of 0.426 (range 0–1), precision score of 0.349 (range 0–1) and recall score of 0.549 (range 0–1) demonstrated low content accuracy. The cosine similarity was 0.609 (range 0–1) and had an average GQS of 3.62 ± 0.49 (range 1–5). These results highlight ChatGPT's suboptimal performance in comparison with the prostate cancer patient information reference sources.^27^ Accordingly there needs awareness for the subtleties of complex and multifactorial medical conditions such as prostate cancer that can be overlooked by AI at this time. However, Gabriel et al. assessed the accuracy of ChatGPT in determining the frequency of complications of robotic‐assisted radical prostatectomy by comparing its responses to the British Association of Urological Surgeons (BAUS) patient information on this topic. Results were graded by two consultant urologists. Of ChatGPT's quoted figures, 11/14 (78.6%) of were concordant and comparable to those on the BAUS patient information pamphlet. ChatGPT's answers were considered to be accurate in 13/14 questions (92.9%).^28^ In comparison, a study by Chiarelli et al. assessed ChatGPT and GPT‐4's responses to prostate cancer screening questions. The LLMs were marked on accuracy, clarity and conciseness on easy, medium and hard questions, as well as readability using Flesch Kincaid Grade (FKG) and Flesch Reading Ease (FRE). ChatGPT's mean score (*SD*) for accuracy, clarity and conciseness was 1.5 (0.59), 1.7 (0.45) and 1.7 (0.49) for easy questions; 1.3 (0.67), 1.6 (0.69) and 1.3 (0.65) for medium questions; 1.3 (0.62), 1.6 (0.56) and 1.4 (0.56) for hard questions, respectively. GPT‐4's mean score was 2.0 (0), 2.0 (0) and 2.0 (0.14) for easy questions; 1.7 (0.66), 1.8 (0.61) and 1.7 (0.64) for medium questions; 2.0 (0.24), 1.8 (0.37) and 1.9 (0.27) for hard questions, respectively. The FKG mean (SD) for ChatGPT and GPT‐4 was 12.8 (1.75) and 10.8 (1.72). The FRE for ChatGPT and GPT‐4 was 37.3 (9.65) and 47.6 (9.88).

The results show that GPT‐4 performed better than ChatGPT in every aspect and was more easily comprehensible to readers.^29^ ChatGPT has been found to be inconsistent in its performance across multiple studies, but GPT‐4 appears to hold greater potential as a successful chatbot in the clinical setting.

The performance of LLMs on European board examinations have been tested by Kollitsch et al. Their group assessed ChatGPT, GTP‐4 and Bing on the 2022 European Board of Urology In‐Service Assessment over multiple rounds of testing. GPT‐4 scored 63%, 77% and 77% while Bing achieved marks of 81%, 73% and 77%, both of which consistently achieved above the pass mark of 61%. However, ChatGPT only obtained scores of 58%, 62% and 59%. All three LLMs exhibited worse scores with increasing complexity of questions.^30^ Deebel et al. also assessed the performance of ChatGPT on American urological examinations. They quizzed ChatGPT on American Urological Association Self‐assessment Study Program questions from 2021 and 2022 and the results showed that ChatGPT performed better on 2021 questions (42.3%) in comparison to 2022 questions (30%).^31^

Other types of chatbots have also been used within urology. These include chatbots for sexually transmitted infections screening; a chatbot for prostate cancer communication called PROSCA about the early detection of prostate cancer; and MenGO, a cloud‐based digital healthcare platform specifically for andrology.^32^

**TABLE 2:** The role of chatbots in surgery.

Roles	Examples
Analysing big data	The PRIME AI framework has been shown to accurately analyse emotions in prostate cancer patients.
Administrative tasks	Answering frequently asked questions about post‐operative care, providing personalised resources to patients and explaining medical concepts in a simplified manner. Writing operation reports and discharge summaries.
Research	Writing grants. Reviewing journal articles. Writing drafts of research papers.
Education	Medical and resident education through generating educational materials on a variety of topics.

### 3.4 Use of chatbots in surgery

Chatbots have been tested in various other surgical specialties as well. In general surgery, studies have assessed ChatGPT's responses to questions in pancreatic cancer and bariatric surgery by experts in their respective fields and with most responses graded as ‘very good’ or ‘excellent’.^33^, ^34^ Writing discharge summaries and operation notes is another area of interest with ChatGPT. Robinson et al. used GPT‐4 to produce operation notes for laparoscopic appendicectomy that were evaluated against ‘Getting it Right First Time (GIRFT) recommendations’ with an average coverage of 78.8% (23.66/30) of the guidelines. ChatGPT has been used in multidisciplinary meetings to test its capabilities in the planning of cancer patients. Lukac et al. showed that ChatGPT was unable to provide specific recommendations for treatment of breast cancer patients and even provided incorrect advice in one instance. The chatbot gave mostly generalised answers on surgery, chemotherapy, radiotherapy and antibody therapy.^35^ In Korea, ChatGPT and GPT‐4 were tested on the general surgery board exams. GPT‐4 was found to perform markedly better with an accuracy of 76.5% compared with ChatGPT (46.8%).^36^

In addition, general surgeons have compared the performance of different types of LLMs. Lee et al. tested ChatGPT, Bard and Bing on frequently asked questions in bariatric surgery using pre‐existing guidelines. Their results showed that GPT‐4 answered the most questions appropriately (85.7%), followed by Bard (74.3%) and Bing (25.7%). They also used 5‐point Likert scores with mean values of 4.46 (*SD* 0.82) for GPT‐4, 3.89 (0.80) for Bard and 3.11 (0.72) for Bing.^37^ Similarly, Huo et al. explored recommendations for surgical management of gastroesophageal reflux disease given by GPT‐4, Copilot, Bard and Perplexity. The chatbot answers were compared with the Society of American Gastrointestinal and Endoscopic Surgeons guidelines. Accurate surgical recommendations for an adult were given in 85.7% of questions by Bard, 71.4% by GPT‐4, 42.9% by Copilot and 42.9% by Perplexity.^38^

Balel et al. in oral and maxillofacial surgery used ChatGPT to examine frequently asked questions asked by patients, and technical questions for training. The answers were evaluated by experienced maxillofacial surgeons who found that ChatGPT scored higher to patient questions by a statistically significant margin.^39^ Hoch et al. also tested ChatGPT on otolaryngology board certification preparation questions, which only answered 57% of questions correctly.^40^ Similarly, Long et al. used GPT‐4 on a selection of questions from the Royal College of Physicians and Surgeons of Canada's sample exam.^41^

In neurosurgery, Haemmerli et al. tested ChatGPT in brain glioma decision making on adjuvant therapy in patient's from a central nervous system tumour board and found that ChatGPT was poor in diagnosing glioma subtypes, but made good adjuvant treatment recommendations. However, once again, it was unable to give specific recommendations and taking into account the patient's functional status.^42^ Furthermore, Ali et al. tested ChatGPT and GPT‐4 on a mock neurosurgery written board exam and GPT‐4 outperformed ChatGPT with scores of 83.4% to 73.4%, respectively.^43^ Similarly, the same group also tested different forms of generative AI on a neurosurgery oral boards preparation question bank that contained predominantly higher‐order questions. GPT‐4 answered 82.6% of questions correctly, ChatGPT scored 62.4% and Bard 44.2% only.^44^

In orthopaedic surgery, ChatGPT was tested on thromboembolic prophylaxis in spine surgery by comparing its responses with the North American Spine Society (NASS) clinical guidelines for antithrombotic therapy. Both ChatGPT‐3.5 and GPT‐4 were used and the categories tested were accuracy, over‐conclusiveness, supplementary information, and incompleteness. ChatGPT‐3.5 was found to be less accurate (4/12 [33%] vs. 11/12 [92%]), more over‐conclusive (6/12 [50%] vs. 1/12 [8%]), gave less supplemental information (8/12 [67%] vs. 11/12 [92%]), and the same number of incomplete responses 4/12 (33%) as GPT‐4.^45^ ChatGPT has also been assessed on orthopaedic examinations in various countries, only scoring 35.8% on one section of the Trauma and Orthopaedic Surgery Fellowship of the Royal College of Surgeons examination from the United Kingdom in a study by Cuthbert et al.^46^ Likewise, Lum used ChatGPT on the Orthopaedic In‐Training Examination in America and found it answered 47% of questions correctly, which was comparable with the level of a first‐year orthopaedic surgery resident.^47^

Plastic surgeons have used ChatGPT on the management of carpal tunnel syndrome using a Likert framework, and it was shown to be able to deliver superficial information, but often had incorrect references.^48^ Two Finnish plastic surgery national board examiners tested LLMs on the national Finnish plastic surgery written examination. They used both ChatGPT and Bing; however, neither of them was able to pass the exam. A minimum score of 15 points is needed to successfully pass the exam, and ChatGPT had a score of 7.5, while Bing scored only five.^49^ In addition, ChatGPT has been applied to research in surgery to generate novel ideas and to write R01 Grant.^50^, ^51^ The standard was not good enough to be a grant awardee but had the potential to help novice researchers.

In ophthalmology, Singh et al. explored ChatGPT's ability in writing discharge summaries and operative notes. The discharge summaries consisted of a significant amount of generic text, and the operative notes required significant tuning despite being detailed. One common theme was the quality of the inputs to ChatGPT often determined the quality of the responses.^52^

Generative AI has been applied to numerous aspects of healthcare by the different surgical specialties. However, there has been shown significant variation between the LLMs used and even between specialties. For instance, ChatGPT's performance on academic examinations has shown mixed results. Educators will need to be aware of the potential for further evolution in this area to potentially disrupt accepted models of surgical assessment of proficiency. This can also be applied to all other areas of surgery as AI is being increasingly utilised within healthcare. As such, it is very much a growing field that will show substantial change over the coming years.

### 3.5 Other forms of generative AI

In terms of other generative AI, significant gains have been made by rival companies to OpenAI in developing their own AI and subsequently integrating it into healthcare. Google has created Med‐PaLM 2, an LLM with the aim of specifically being used for the medical domain. Med‐PaLM 1 and 2 have made remarkable progress in successfully passing the United States Medical Licencing Examination with scores of 67.6% and 86.5%, respectively.^53^ Microsoft has focused heavily on healthcare and identified AI in healthcare as a key area of growth. It acquired the technology company Nuance, which created the AI‐powered transcription platform now called Dragon Ambient eXperience (DAX) Copilot in 2021. Consisting of a user base of over 500 000 physicians, DAX Copilot automatically drafts summaries of clinic consultations in real time that is available for physician review in seconds and subsequent entry in an electronic health record (EHR) system.^54^ Similarly, Microsoft has also collaborated with Epic, a major EHR company in America, into integrating generative AI into its software. This includes summarising patient information from clinical charts, drafting patient letters and AI suggesting medical code to streamline coding and billing processes.^55^ Moreover, DALL‐E 2, an AI‐powered text‐to‐image generator developed by OpenAI, has been shown to be able to generate accurate normal x‐ray images, but was poor in the generation of pathology. Other imaging modalities including CT, MRI and ultrasound (US) were poorly generated by DALL‐E 2. This is not useful to urology in its current form, where common urological scans include CT intravenous pyelography (IVP), MRI prostate and US kidney, ureter and bladder. These developments represent a significant step forward in generative AI changing clinical practice in the near future, which will benefit time‐poor urologists, one of the busiest specialties and allow them to focus greater energy on the more important clinical aspects of their workload.^56^

### 3.6 Our ChatGPT experience

In the preparation of this manuscript we also asked ChatGPT about what it could be used for in surgery, and urology in particular. The prompt used was ‘write a review on ChatGPT and its uses in urology’. It additionally suggested that ‘ChatGPT has the ability to process large amounts of data quickly and accurately, and can help urologists to make more informed decisions about patient care, by analyzing patient data and identifying patterns that might not be immediately apparent to human clinicians. This can help to identify patients who are at high risk of developing urological conditions. By analyzing patient data and medical records, ChatGPT can help to identify potential causes of symptoms and suggest appropriate diagnostic tests, reducing the time it takes to make a diagnosis.’.^57^

Interestingly when providing ChatGPT with specific instructions to ‘write a narrative review on ChatGPT in surgery of 2000‐4000 words following the BJUI format with Vancouver references. Also include a 300 word abstract with introduction, methods, results and conclusion’, it was unable to comply, replying with ‘generating a full narrative review with references and adhering to a specific format like the BJUI (British Journal of Urology International) is an extensive task that goes beyond the scope of a single response. Additionally, I cannot generate specific Vancouver references. However, I can help you outline the narrative review and provide an abstract that you can use as a starting point. If you'd like, I can also assist you in generating specific sections of the review’. It then provided a 238‐word abstract (see supporting information).

### 3.7 Cautions in using ChatGPT

ChatGPT, like with any LLM, is prone to errors and mistruths but conveys its responses in a confident and authoritative tone that makes it appear believable due to the way the model is built. This phenomenon is known as artificial hallucination^58^ and has the risk of misleading, misinterpreting and spreading misinformation, which can be particularly dangerous in the healthcare setting. Bias is another potential issue of concern, where the internet content upon which ChatGPT was trained has its own inherent biases, which in turn will only be recited by ChatGPT. AI software is only as good as the data it is built upon. Patient confidentiality and privacy is an additional major concern in healthcare that may also be problematic for the use of ChatGPT in a healthcare setting where any confidential patient data fed into ChatGPT will be stored on its cloud servers, where the extent of future privacy protection is unknown.^59^, ^60^

## 4 CONCLUSION

The potential of ChatGPT is vast, and novel users of this platform often are left with the sense that this is the beginning of a new era for AI, where this may eventually become integrated seamlessly into surgical practice just as email and electronic medical records have done. The further development of this and other generative AI offers potential for incorporation into surgical workflow in numerous forms. Concerns with this new technology however must not be disregarded in the urge to hasten this progression, and potential risks impacting against the best interests of patients must be weighed carefully against the benefits. Appropriate governance of the use of this technology will be the key to optimising outcomes, and it is incumbent upon urologists to commence to create appropriate safeguards now, that will serve patients and clinicians judiciously for the foreseeable future.^61^

## AUTHOR CONTRIBUTIONS

**Shane Qin:** Writing the original draft; visualisation. **Bodie Chislett:** Writing the original draft. **Joseph Ischia:** Conceptualisation; supervision. **Weranja Ranasinghe:** Writing review and editing. **Daswin de Silva:** Writing review and editing. **Jasamine Coles‐Black:** Writing review and editing. **Dixon Woon:** Writing review and editing. **Damien Bolton:** Conceptualisation, supervision; writing review and editing.

## CONFLICT OF INTEREST STATEMENT

No conflicts of interest.

## Supporting information

Supporting Information S1
